# Alleviation Effects of Microbial Metabolites from Resveratrol on Non-Alcoholic Fatty Liver Disease

**DOI:** 10.3390/foods12010094

**Published:** 2022-12-24

**Authors:** Jingling Guo, Pan Wang, Yifan Cui, Xiaosong Hu, Fang Chen, Chen Ma

**Affiliations:** 1 National Engineering Research Center for Fruit and Vegetable Processing, Key Laboratory of Fruits and Vegetables Processing, Ministry of Agriculture, Engineering Research Centre for Fruits and Vegetable Processing, Ministry of Education, College of Food Science and Nutritional Engineering, China Agricultural University, Beijing 100083, China; 2 Beijing Key Laboratory of Agricultural Products of Fruits and Vegetables Preservation and Processing, Key Laboratory of Vegetable Postharvest Processing, Ministry of Agriculture and Rural Affairs, Institute of Agri-Food Processing and Nutrition, Beijing Academy of Agriculture and Forestry Sciences, Beijing 100097, China

**Keywords:** NAFLD, microbial metabolites, 3-hydroxyphenyl propionic acid, 4-hydroxyphenyl propionic acid, lipid metabolism

## Abstract

Resveratrol (RSV), a polyphenolic stilbene, has been widely studied for its protective effects against non-alcoholic fatty liver disease (NAFLD) by modulating intestinal microbiota. The microbial metabolites after RSV supplement would contribute to the bioeffects of RSV, while their impacts on NAFLD were unclear. Therefore, this study aimed to investigate the beneficial effects of the main microbial metabolites from RSV on lipid metabolism by combining in vitro and in vivo models. The mice were fed a high-fat diet and injected with RSV, 3-hydroxyphenyl propionic acid (3-HPP), and 4-HPP for 13 weeks (*n* = 6). Body weight, serum parameters, histological analysis, and gene expression involved in lipid metabolism were quantified. Our results suggested that 100 μM of 3-HPP and 4-HPP inhibited lipid accumulation more significantly than parent RSV in an oleic acid-induced HepG2 cell line. Furthermore, 3-HPP, 4-HPP, and RSV effectively reduced liver weight and body weight, improved hepatic steatosis, and alleviated systemic inflammation in NAFLD mice. In addition, the results of quantitative real-time PCR showed that 3-HPP and 4-HPP altered the expression of cholesterol influx and efflux genes to a stronger extent than RSV. These results indicate that 3-HPP and 4-HPP are effective in regulating hepatic lipid metabolism.

## 1. Introduction

Non-alcoholic fatty liver disease (NAFLD) is a chronic liver disease affecting 25% of adults and 3–10% of children globally [1,2]. NAFLD is manifested by hepatic steatosis, inflammation, gut microbiota dysbiosis, and insulin resistance without an alcoholic abuse history and can even progress to steatohepatitis, fibrosis, and hepatocellular carcinoma [3]. The imbalance between neutral lipid acquisition (biosynthesis and import) and neutral lipid disposal (conversion, degradation, and export) plays a vital role in hepatic steatosis [4]. Moreover, NAFLD is strongly associated with multiple epidemics [5,6,7], such as obesity and cardiovascular disease, which pose a heavy economic burden on patients [8]. Until now, there is no drug for treating NAFLD clinically.

Resveratrol (RSV) is a polyphenolic stilbene, which is widely present in grapes, berries, and peanuts [9]. Previous evidence has suggested that RSV can improve NAFLD in preclinical studies [10,11]. The molecular mechanism on alleviating NAFLD by RSV majorly contains regulating lipid metabolism, ameliorating inflammation, decreasing insulin resistance, and modulating gut microbiota composition [12,13,14,15]. As the main treatment mechanism, RSV can decrease hepatic lipid levels by modulating the activity of fatty acid (FA) oxidation and transport proteins, such as carnitine palmitoyltransferase 1a (CPT1a), FA transport protein 5 (FATP5), and cluster of differentiation 36 (CD36) [16,17,18]. Nevertheless, the improvement of NAFLD by RSV is strikingly in contrast to its bioavailability (less than 1%) [19].

Gut microbiota, a virtual endocrine organ, can transform polyphenols into small molecular metabolites that increase the bioavailability of polyphenols [20]. Recent works demonstrated that the bioactivities of RSV depended on gut microbiota according to the results of fecal microbiota transplantation [15,21,22,23]. Therefore, we assumed that the beneficial effects of RSV on lipid metabolism were mediated through microbial metabolites. The microbial metabolites of RSV mainly included dihydroresveratrol (DHR), 3,4′-dihydroxystilbene, lunularin (LUN), 4-hydroxydibenzyl, and phenolic acids such as 3- and 4-hydroxybenzoic acid (3-HBA, 4-HBA), 3- and 4-hydroxyphenylpropionic acid (3-HPP, 4-HPP), and 4-hydroxyphenylacetic acid (4-HPA) [24,25,26,27]. Gunther et al. found that DHR and LUN supplementation had little influence on body composition of high-fat diet (HFD)-fed mice [28]; which was inconsistent with in vitro findings [29,30,31]. Moreover, the effects of phenolic acid metabolites from RSV on lipid metabolism have rarely been investigated.

Interestingly, except for resveratrol, procyanidins, epicatechin, and other flavanols can also be transformed into these phenolic acids by gut microbiota [32,33,34]. In recent years, phenolic acid metabolites of polyphenols have gradually received attention because of their beneficial effects [35,36]. 3,4-dihydroxyphenylacetic acid (DHPAA) and 2,3-dihydroxybenzoic acid (DHBA) are the microbial-derived flavonoid metabolites, but their bioactivities are different. DHPAA played protective roles on insulin signaling blockade and oxidative stress while DHBA prevented lipopolysaccharide-induced inflammation [37,38,39]. In addition, 3,4-dihydroxytoluene, a metabolite of rutin, was capable of ameliorating hepatic lipid accumulation by inhibiting histone acetyltransferase activity [40]. Moreover, protocatechuic acid, a microbial metabolite of anthocyanin, can regulate lipid metabolism, oxidative stress, intestinal microbial homeostasis, and inflammation, thereby exhibiting protective effects against NAFLD [41].

Therefore, this work aimed to assess the efficacy of microbial phenolic acids after RSV supplementation on NAFLD using an oleic acid (OA)-induced cell model and an HFD-induced mice model. Our findings suggest that 3-HPP and 4-HPP can attenuate hepatic steatosis and systemic inflammation, and their effects were the same as those of RSV. Interestingly, 3-HPP and 4-HPP mainly influenced cholesterol transport, while RSV primarily affected FA oxidation and transport.

## 2. Materials and Methods

### 2.1. Materials

RSV, 3-HBA, 3-HPP, 4-HBA, 4-HPA, and 4-HPP (purity > 95%) were purchased from Aladdin, Shanghai, China. The sodium salt of OA was obtained from J&K Scientific, Beijing, China. Phosphate buffered saline (PBS), polyethylene glycol 400, Cell Counting Kit-8 (CCK8), the BCA Protein Assay Kit, and Oil Red O (ORO) solution were obtained from Solarbio, Beijing, China.

### 2.2. Cell Culture

The human hepatocellular carcinoma cell line HepG2 was gifted by Professor Hongbo Hu (China Agricultural University, Beijing, China). The cells were cultured in DMEM containing a 1% supplement of antibiotic solution (10,000 IU/mL penicillin and 10 mg/mL streptomycin) and 10% fetal bovine serum in an incubator with constant H_2_O vapor pressure (5% carbon dioxide, 37 °C). When the cell confluence approached 70%, the cells could be passaged. Cultures between the 4th and 10th passages were used for different experiments.

OA mother solution (100 mM) was prepared by dissolving sodium salt of OA powder in double distilled water at 70–75 °C. It was then combined with 15% (*w*/*v*) BSA solution to obtain the OA stock (10 mM/10% BSA). The stock was filtered and diluted with DMEM to obtain the OA working solution (1 mM OA/1% BSA). The NAFLD cell model was induced using the working solution, which was treated with HepG2 cells for 24 h. As a control, 1% BSA solution was used to treat cell cultures. HepG2 cells were then treated with RSV and phenolic acids and the OA working solution for 24 h. 

### 2.3. CCK8 Assay

HepG2 cells were seeded in 96-well plates with a proper density (100,000 cells/mL). After 12–14 h, the cells were treated with various concentrations (25–200 μM) of RSV and phenolic acids in the absence and presence of the OA working solution for 24 h. The cells were then treated with 10% CCK8 reagent solution for 1 h in a dark environment. Following this, the absorbance was measured at 450 nm wavelength.

### 2.4. Measurement Triglyceride (TG) Level of HepG2 Cells

HepG2 cells were seeded in 60 mm dishes. The cells were treated with 1% BSA solutions (C group), the OA working solution (OA group), the OA working solution with RSV (RSV group), and the OA working solution with phenolic acids (phenolic acids group) for 24 h. After these treatments, the cells were collected and then crushed with PBS by using an ultrasonic cell crusher (Scientz, Ningbo, China). Then, the protein concentration of the crushed liquid was measured with the BCA Assay Kit to normalize the number of cells and the TG level in the cells was measured using the TG Assay Kit (Jiancheng, Nanjing, China).

### 2.5. ORO Staining of HepG2 Cells

HepG2 cells were seeded in 35 mm dishes with slides. The cells were treated with 1% BSA solutions (C group), the OA working solution (OA group), the OA working solution with RSV (RSV group), and the OA working solution with phenolic acids (phenolic acids group) for 24 h. After these treatments, the cells were washed and fixed for 25–40 min. Following this, these were washed and stained with 60% (*v*/*v*) ORO solution in the dark for 1 h. Lastly, the cells were sealed with pure water to observe and photograph under an inverted microscope (Nikon, Tokyo, Japan) at 400× magnification. The results of ORO staining were semi-quantified using Image-Pro Plus 6.0.

### 2.6. Feeding and Grouping of Mice

The study protocol was approved by the China Agricultural University Laboratory Animal Welfare and Animal Experimental Ethics Committee (Beijing, China) (No. AW70702202-4-1). A total of 30 4-week-old male C57BL/6J mice (16–18 g) were bought from Beijing Vital River Laboratory Animal Technology Co Ltd., China. After 7 days of adaptive feeding in a barrier environment (10–14 h light/dark cycle, 22 ± 2 °C, 55 ± 5% humidity), mice were kept in cages (three mice per cage) and classified into 5 groups with 6 mice in each group: the normal diet (ND), HFD, RSV, 3-HPP, and 4-HPP group.

For 13 consecutive weeks, the ND group was supported with a control diet (Research Diets, New Brunswick, NJ, USA; D12450J) and the other groups were fed HFD (Research Diets, New Brunswick, NJ, USA; D12492). The macronutrient composition of the two diets is shown in the Supplementary Materials, Table S2. For intraperitoneal injection (i.p.), 3-HPP, 4-HPP, and RSV were dissolved in PEG400 and then diluted with saline to obtain 20% (*v*/*v*) PEG solutions. The mice in the RSV, 3-HPP, and 4-HPP groups were intraperitoneally injected with the PEG solutions at a dose of 24 mg/kg BW twice a week. The mice of the ND and HFD groups were intraperitoneally injected with 20% PEG with saline.

On the 89th d, the mice were fasted for 16 h, weighed, and sacrificed. Following this, we collected their blood, white adipose tissue (epididymal fat, inguinal fat, and perirenal fat), and liver. The serum samples were acquired by centrifuging blood for 25 min at 3300 rpm at 4 °C. After weighing, a piece of epididymal fat and a piece of liver tissue that were approximately 1 cm^3^ in size were fixed with 4% paraformaldehyde at a ratio of 10:1 for 24 h. The remaining samples were loaded into cryovials, placed in dry ice, and subsequently stored at −80 °C.

### 2.7. Intraperitoneal Glucose Tolerance Test (IPGTT)

On the 82nd d, the mice were fasted for 16 h. Following this, the mice were intraperitoneally injected with 20% (*w*/*v*) glucose solution (2.0 g/kg BW). Blood samples were collected from the tail vein at several time points, and blood glucose levels were measured with glucometers (Active, Roche, Switzerland). 

### 2.8. Biochemical Analysis of Serum and Liver Samples

Test kits (Jiancheng, Nanjing, China) were selected to assess the serum levels of aspartate aminotransferase (AST), alanine aminotransferase (ALT), total cholesterol (TC), TG, low-density lipoprotein cholesterol (LDL-c), HDL-c, and the hepatic TG and TC levels. The serum levels of insulin, interleukin-6 (IL-6), IL-8, and tumor necrosis factor-α (TNF-α) were measured using ELISA kits (Genelab, Beijing, China). Homeostatic model assessment for insulin resistance (HOMA-IR) was calculated according to a previous study [42]. 

### 2.9. Histopathological Examination

Fixed epididymal fat and liver were embedded with optimal cutting temperature compound and quickly frozen. When the specimen became white and hard, it was sectioned (8–10 μm) and dyed with hematoxylin and eosin (H&E) or ORO and hematoxylin. Staining results were visualized under an inverted microscope at 200× magnification and photographed using a digital camera. The results of H&E staining of liver tissues were scored on the basis of the NAFLD Activity Score (NAS) [43]. Histological features were scored on a scale of 0–3 for steatosis, lobular inflammation, and ballooning. NAFLD was considered to be present when the total score was above 4 and absent when the total score was less than 3. The graphs of ORO staining of liver tissues and H&E staining of epididymal fat samples were semi-quantified using Image-Pro Plus 6.0 and Image J.

### 2.10. RNA Isolation and qPCR Analysis

Liver samples were pre-treated by liquid nitrogen milling and then total RNA was isolated. The concentration and purity of RNA were then assessed by detecting OD230, OD260, and OD280 using NanoDrop 2000 (Thermo Fisher, Darmstadt, Germany). High-purity RNA samples were converted into cDNA. Quantitative real-time polymerase chain reaction (qPCR) analysis was performed using the BioRad CFX96 System. The primer sequences (Tsingke Biotechnology, Beijing, China) are listed in Table 1 and their amplification efficiency is shown in the Supplementary Materials, Table S1. Finally, the expression of target genes was standardized to the *gapdh* expression and estimated with the 2^−∆∆Ct^ method. 

### 2.11. Statistical Analysis

Data are expressed as the means ± standard errors (SEMs). SPSS 20.0 was selected to compare statistically and GraphPad Prism 8 was used to show the results. All experiments were repeated 3 times in this work. One-way analysis of variance (ANOVA) was used to assess the significant difference among the 5 groups. *p* < 0.05 was considered markedly different in accordance with Duncan’s test.

## 3. Results

### 3.1. Phenolic Acids Reduced Lipid Contents in OA-Induced NAFLD Cell Model

**Figure 1:**
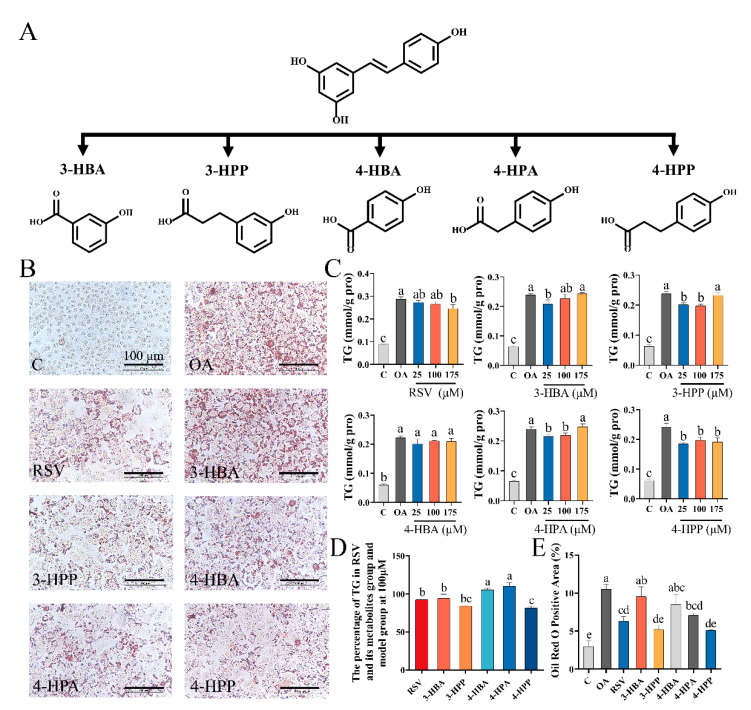
Effects of resveratrol (RSV) and phenolic acids on lipid content of HepG2 cells. (**A**) Chemical structure of RSV and phenolic acids. (**B**) Images of oil red oil (ORO) staining of HepG2 cells after treatment with 100 μM of RSV and phenolic acids. (**C**) Effects of RSV and phenolic acids at 25 μM, 100 μM, and 175 μM on triglyceride (TG) levels in HepG2 cells. (**D**) Comparison of the effects of 100 μM of RSV and phenolic acids on reducing TG levels. (**E**) The red area ratio of lipid droplets in ORO staining graphs was semi-quantified with Image-Pro Plus 6.0. Data without a common letter indicate marked differences based on Duncan’s test when *p* < 0.05.

The molecular structures of phenolic acids and RSV are shown in Figure 1A; their effects on the accumulation of hepatic lipids were assessed. HepG2 cells were incubated with increasing concentrations of RSV and phenolic acids (25–200 μM) with and without OA for 24 h. Following this, cell viability was assessed. RSV treatment caused no discernible toxicity in cells even at 175 μM (Supplementary Materials, Figure S1). The effects of RSV and phenolic acids on lipid accumulation induced by OA were assessed based on TG levels and ORO staining results. Treatment with RSV, 3-HBA, 3-HPP, and 4-HPP at 100 μM reduced the TG levels by 8.55%, 6.94%, 16.87%, and 18.52%, respectively (Figure 1C,D); this was accompanied by a decrease in lipid droplets (Figure 1B,E). Moreover, 3-HPP and 4-HPP were more efficacious than RSV in inhibiting the accumulation of lipids. Therefore, the effect of 3-HPP, 4-HPP, and RSV on NAFLD was further assessed using an in vivo model.

### 3.2. 3-HPP and 4-HPP Had Little Influence on Lipid Anabolism in Adipocytes of HFD Mice

**Figure 2:**
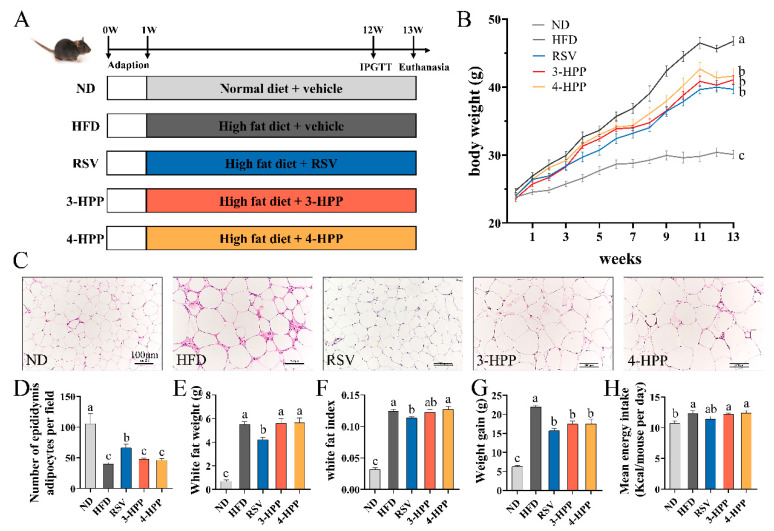
Effects of RSV, 3-hydroxyphenyl propionic acid (3-HPP), and 4-HPP on lipid accumulation and anabolism in white adipose tissue of HFD mice. (**A**) Schematic diagram of animal experiment. (**B**) Body weight changes over 13 weeks. (**C**) Morphological examination of epididymal fat by hematoxylin and eosin (H&E) staining (*n* = 3, 200×). (**D**) The number of adipocytes in epididymis per field. (**E**) White fat weight. (**F**) White fat index. (**G**) Gain of body weight. (**H**) Mean energy intake. Data are expressed as the means ± SEMs. Data without a common letter indicate marked differences in line with Duncan’s test when *p* < 0.05. *n* = 6 if not specified. White fat includes epididymal fat, inguinal fat, and perirenal fat. ND, normal diet; HFD, high-fat diet; RSV, high-fat diet and i.p. with 24 mg/kg BW RSV; 3-HPP, high-fat diet and i.p. with 24 mg/kg BW 3-HPP; 4-HPP, high-fat diet and i.p. with 24 mg/kg BW 4-HPP.

Five-week-old C57BL/6J mice were supported with an ND or HFD (Figure 2A). The mice were intraperitoneally injected with vehicle, 3-HPP, 4-HPP, or RSV. During this 13-week period, HFD significantly induced body weight gain in the mice, while 3-HPP, 4-HPP, and RSV reduced it without any change in the energy intake (Figure 2B,G,H). HFD mice had more white fat and a higher white fat index. Compared with RSV supplementation, 3-HPP and 4-HPP supplementation failed to reduce white fat weight and white fat index; this result was consistent with that of histological analysis of epididymal fat by H&E staining (Figure 2C–F). These results suggest that 3-HPP and 4-HPP were unable to reduce lipid anabolism in white adipose tissue.

### 3.3. 3-HPP and 4-HPP Alleviated Liver Damage and Hepatic Steatosis in HFD Mice

**Figure 3:**
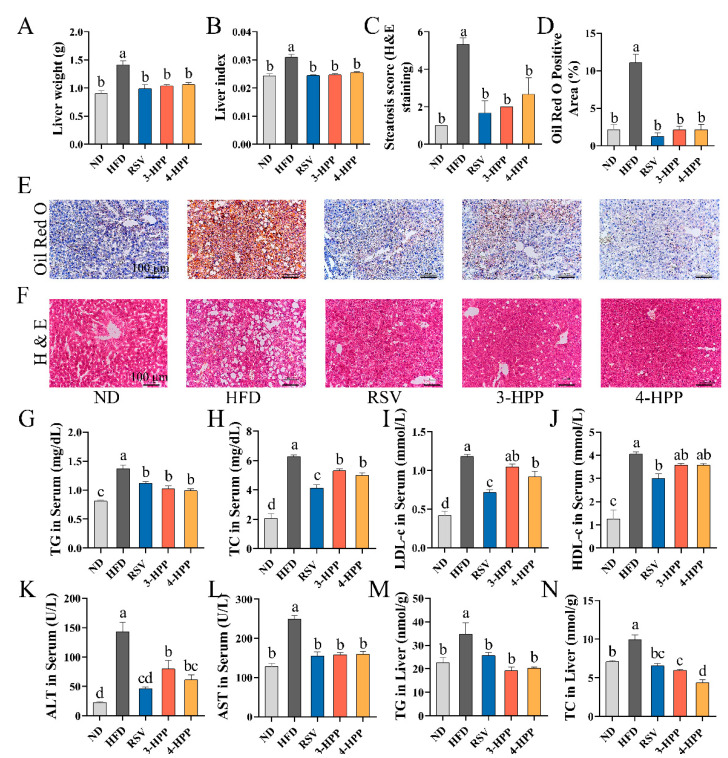
Effects of RSV, 3-HPP, and 4-HPP on hepatic and serum lipid profiles of HFD mice. (**A**) Weight of liver. (**B**) The ratio of liver weight to body weight. (**C**) NAFLD Activity Score (NAS) of H&E staining (*n* = 3). (**D**) Lipid droplet area ratio in ORO staining graphs (*n* = 3). (**E**) Morphological examination of the liver sample by H&E staining (*n* = 3, 200×). (**F**) Hepatic lipid droplet visualization by ORO staining (*n* = 3, 200×). (**G**) Level of serum TG. (**H**) Level of serum total cholesterol (TC). (**I**) Level of serum low-density lipoprotein cholesterol (LDL-c). (**J**) Level of serum high-density lipoprotein cholesterol (HDL-c). (**K**) Level of serum alanine aminotransferase (ALT). (**L**) Level of serum aspartate aminotransferase (AST). (**M**) Hepatic TG level. (**N**) Hepatic TC level. Data are expressed as the means ± SEMs. Data without a common letter indicate marked differences based on Duncan’s test when *p* < 0.05. *n* = 6 if not specified. ND, normal diet; HFD, high-fat diet; RSV, high-fat diet and i.p. with 24 mg/kg BW RSV; 3-HPP, high-fat diet and i.p. with 24 mg/kg BW 3-HPP; 4-HPP, high-fat diet and i.p. with 24 mg/kg BW 4-HPP.

After 13 weeks, compared with ND, HFD significantly increased liver weight and liver index; however, 3-HPP, 4-HPP, and RSV supplementation reduced liver weight and liver index (Figure 3A,B). Then, serum and hepatic levels of parameters indicative of lipid accumulation and liver injury were assessed. Compared with HFD, supplementation with 3-HPP, 4-HPP, and RSV significantly reduced hepatic TC and TG levels and ALT, AST, TG, TC, and LDL-c contents in serum (Figure 3G–N). Moreover, histological analysis revealed that HFD induced histological features of NAFLD, such as lipid droplet accumulation, lipid vacuole formation, ballooning, and lobular inflammation. Interestingly, 3-HPP, 4-HPP, and RSV supplementation markedly ameliorated these histological features to a similar level to ND mice (Figure 3C–F).

### 3.4. 3-HPP and 4-HPP Improved Systemic Inflammation and Insulin Resistance in HFD Mice

**Figure 4:**
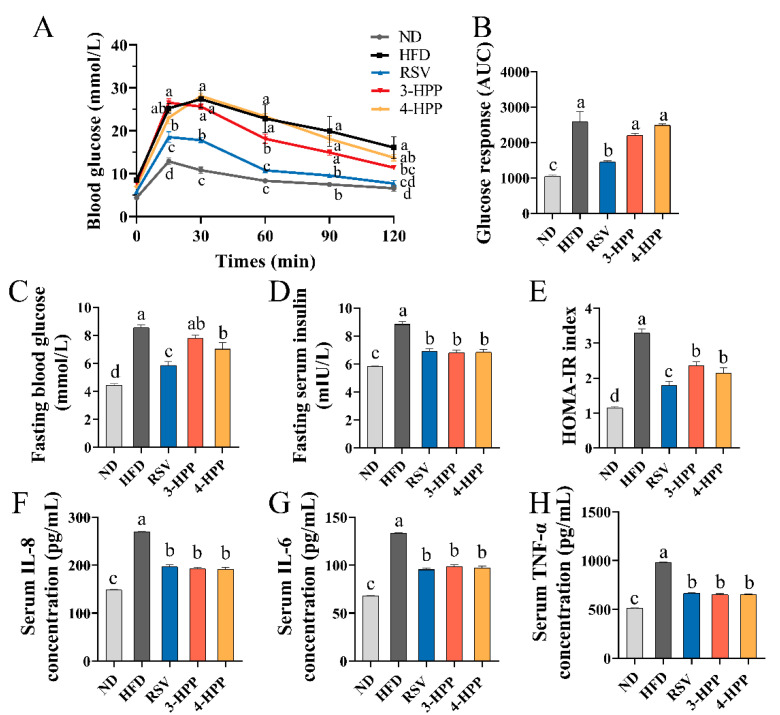
Effects of RSV, 3-HPP, and 4-HPP on insulin resistance and systemic inflammation. (**A**) The intraperitoneal glucose tolerance test (IPGTT) curve. (**B**) Area under the IPGTT curve. (**C**) The level of blood glucose after fasting. (**D**) The level of insulin after fasting. (**E**) An index based on homeostatic model assessment for insulin resistance (HOMA-IR). (**F**) Level of interleukin-8 (IL-8) in serum. (**G**) Serum IL-6 level. (**H**) Tumor necrosis factor-α (TNF-α) level in serum. Data are expressed as the means ± SEMs. Data without a common letter indicate marked differences in accordance with Duncan’s test when *p* < 0.05. *n* = 6 if not specified. ND, normal diet; HFD, high-fat diet; RSV, high-fat diet and i.p. with 24 mg/kg BW RSV; 3-HPP, high-fat diet and i.p. with 24 mg/kg BW 3-HPP; 4-HPP, high-fat diet and i.p. with 24 mg/kg BW 4-HPP.

Glucose homeostasis and inflammation have been reported to induce NAFLD [44]. Compared with ND mice, HFD mice exhibited increased blood glucose levels, HOMA-IR index, and insulin levels after fasting. The results revealed that 3-HPP, 4-HPP, and RSV supplementation ameliorated HOMA-IR index and insulin levels (Figure 4C–E). However, areas under the blood glucose curve after a glucose bolus in the 3-HPP and 4-HPP groups were parallel to that in the HFD group, indicating that 3-HPP and 4-HPP were unable to improve glucose tolerance (Figure 4A,B). Furthermore, the levels of proinflammatory cytokines (TNF-α, IL-8, and IL-6) were markedly higher in mice serum of the HFD group than the ND group. 3-HPP, 4-HPP, and RSV supplementation reduced the levels of proinflammatory cytokines, suggesting that RSV, 3-HPP, and 4-HPP can effectively ameliorate systemic inflammation (Figure 4F–H).

### 3.5. 3-HPP and 4-HPP Regulated the Gene Expression of Lipid Metabolism in HFD Mice

**Figure 5:**
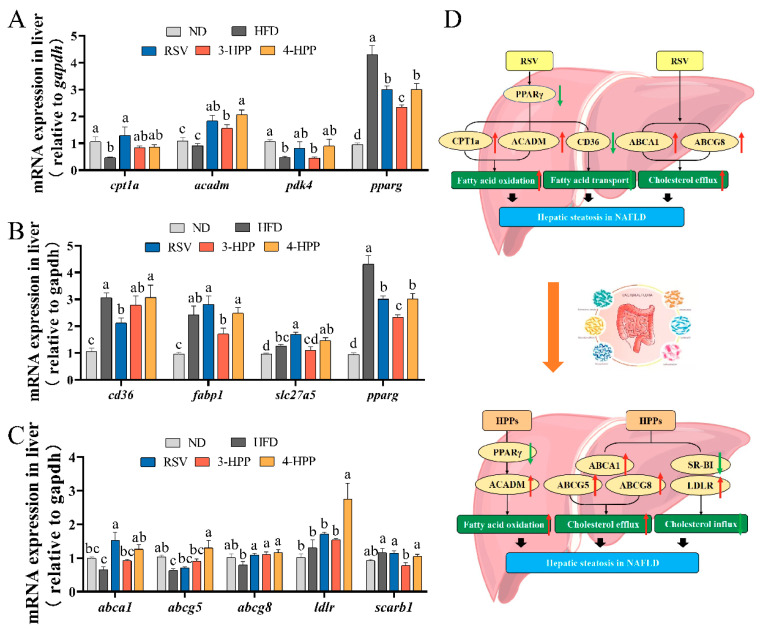
Effects of RSV, 3-HPP, and 4-HPP on hepatic lipid metabolism. (**A**) Hepatic mRNA expression levels of FA transport genes. (**B**) FA β oxidation gene expression in liver. (**C**) Hepatic transcriptional levels of cholesterol transport gene. (**D**) Mechanism underlying the alleviation effects of RSV, 3-HPP, and 4-HPP on HFD diet-induced hepatic steatosis. Data are expressed as the means ± SEMs. Data without a common letter indicate marked differences according to Duncan’s test when *p* < 0.05. *n* = 5 if not specified. ND, normal diet; HFD, high-fat diet; RSV, high-fat diet and i.p. with 24 mg/kg BW RSV; 3-HPP, high-fat diet and i.p. with 24 mg/kg BW 3-HPP; 4-HPP, high-fat diet and i.p. with 24 mg/kg BW 4-HPP.

The expression levels of genes in β oxidation of FA, FA transport, and cholesterol transport were assessed. In Figure 5B, the HFD group exhibited obviously upregulated mRNA expression of genes in FA transport (*cd36*, *fabp1*, and *slc27a5*). Interestingly, only RSV supplementation downregulated the expression of *cd36*, illustrating that 3-HPP and 4-HPP had little influence on FA transport. Moreover, compared with ND, long-term HFD reduced the gene expressions of FA β oxidation (*acadm*, *cpt1a*, and *pdk4*). RSV, 3-HPP, and 4-HPP upregulated the expression of *acadm*, while only RSV upregulated the expression of *cpt1a* (Figure 5A). The expression levels of *abca1*, *abcg5*, and *abcg8*, markers of cholesterol export, were downregulated in HFD mice. Whereas, the gene expression of cholesterol export, such as *ldlr* and *scarb1*, was not significantly influenced by HFD. RSV supplementation upregulated the expression of *abca1* and *abcg8*, 3-HPP supplementation influenced the expression of *abcg8* and *scarb1*, and 4-HPP administration upregulated the expression of *abca1*, *abcg5*, *abcg8*, and *ldlr* (Figure 5C). These results suggest that 4-HPP had the maximum influence on cholesterol export and import in our study.

## 4. Discussion

Increasing evidence has shown that RSV treatment has protective effects on NAFLD by modulating gut microbiota [45]. Intriguingly, RSV treatment influenced gut microbiota structure and stimulated gut microbiota to produce phenolic acids [26]. These small metabolites can enter systemic circulation and influence host health [27,36,46]. Hence, we assessed whether the microbiota-derived phenolic acids of RSV could alleviate NAFLD. In this study, we assessed the effects of 3-HPP, 4-HPP, and RSV by combining in vitro and in vivo models to understand their bioactivity and molecular mechanism.

In the OA-induced HepG2 cell line, 3-HPP and 4-HPP were more effective in reducing lipid accumulation than RSV. By contrast, 3- HBA and 4-HBA did not affect lipid contents and 4-HPA had a weak lipid-lowering activity in HepG2 cells. This result suggests that the molecular structure of metabolites might be related to their effects on lipid accumulation and for the same benzene ring substitution, the length of the carboxyl chain had greater impacts on lipid-lowering activity than the position of the hydroxyl group. As a previous study indicated, the chemical structure of phenolic acids influences their antimicrobial potential [47].

In NAFLD mice, intraperitoneal supplementation with 3-HPP and 4-HPP can alleviate hepatic steatosis, as indicated by liver weight, lipid contents in the liver and serum, and NAS. Surprisingly, the effects of 3-HPP and 4-HPP were the same as those of RSV. While DHR and LUN had little impact on body weight, adipose tissue mass and blood parameters in HFD-fed mice [25]. Our findings elucidated a distinct modulating effect of 3-HPP and 4-HPP on lipid accumulation, which was of significance for treating the problem of beneficial bioactivity and low bioavailability of RSV.

The liver is an essential organ for lipid metabolism. NAFLD primarily occurs as a result of gradually increased lipid accumulation. TG and TC are the major neutral lipids in the liver synthesized by FA, and hepatic steatosis results from an influx and outflux imbalance among these substances in hepatocytes [4]. 3-HPP, 4-HPP, and RSV supplementation significantly lowered lipid levels in the liver and serum, suggesting that RSV and hydroxyphenyl propionic acids can alleviate hepatic steatosis by affecting the acquisition and disposal of major hepatic lipids. The expression levels of FA transport, FA oxidation, and TC transport genes were markedly affected by 3-HPP, 4-HPP, and RSV supplementation. FA transport mainly relies on transport proteins, such as CD36, hepatic FA-binding protein 1 (FABP1), and FATP5 [48]. Interestingly, RSV reduced the upregulation of *cd36* mRNA levels, while 3-HPP and 4-HPP did not influence the expression levels of marker genes of FA transport. Therefore, RSV had the ability to regulate FA transport, which was consistent with the effect of RSV supplementation with oral gavage [13]. CPT1a, medium-chain aryl-CoA dehydrogenase (MCAD), and pyruvate dehydrogenase kinase 4 (PDK4) are key enzymes that are directly involved in FA β oxidation and are regulated by peroxisome proliferator-activated receptor γ (PPARγ) [49]. Compared with HFD, 3-HPP, 4-HPP, and RSV supplementation upregulated *acadm* mRNA levels and downregulated *pparg* mRNA levels. Moreover, RSV supplementation stimulated the expression of *cpt1a*, which was inhibited by HFD; this finding is consistent with previous findings [50]. Scavenger receptor class B type I (SR-BI) and low-density lipoprotein receptor (LDLR) are key mediators of TC absorption from blood and ATP-binding cassette subfamily A member 1 (ABCA1), ABCG5, and ABCG8 are mediators of TC efflux [51]. We found that RSV supplementation upregulated the expression of *abca1* and *abcg8*, 3-HPP supplementation upregulated the expression of *abcg8* and downregulated the expression of *scarb1*, and 4-HPP supplementation increased the expression of *abca1*, *abcg5*, *abcg8,* and *ldlr*. This finding indicates that the bioeffects of 3-HPP and 4-HPP on TC transport are stronger than RSV because RSV did not regulate genes of TG absorption while 3-HPP and 4-HPP did regulate. Importantly, RSV exhibited beneficial effects on lipid metabolism by influencing FA oxidation and transport, while 3-HPP and 4-HPP exhibited beneficial effects by influencing TC transport. After i.p, the majority of RSV and 3-HPP, 4-HPP were directly entered into the blood, while oral administration RSV was metabolized and entered systemic circulation together with its metabolites. Our findings help to illustrate the diverse biological activities of RSV oral administration. 

Besides hepatic steatosis, the development of NAFLD was highly associated with insulin resistance, chronic inflammation, and oxidative stress. We also found that HFD induced insulin resistance and systemic inflammation, which were reversed by 3-HPP, 4-HPP, and RSV supplementation. However, the effects of 3-HPP and 4-HPP on insulin resistance were weaker than those of RSV, which inspired us to find the real and active form in RSV decreasing insulin resistance. Finding this form would be of importance for preventing diabetes by RSV. In addition, 3-HPP and 4-HPP supplementation decreased systemic inflammation as well as RSV supplementation. Future studies could focus on antioxidant enzyme levels in the liver to assess the effects of 3-HPP and 4-HPP on oxidative stress. Moreover, the mechanisms by which 3-HPP and 4-HPP inhibit inflammation need to be explored in future studies.

## 5. Conclusions

In conclusion, we found that 3-HPP and 4-HPP, gut microbiota-derived metabolites of RSV, can alleviate NAFLD in OA-induced HepG2 cells and HFD-fed mice by ameliorating hepatic steatosis and their bioeffects were equivalent to RSV at the same concentration. While the molecular mechanisms between the parent compound and microbial metabolites on regulating lipid metabolism are different, the former focuses on FA transport and the latter focuses on cholesterol transport. Our finding helps to explain the contradiction between low bioavailability and beneficial effects of RSV and other polyphenols. Furthermore, our study illustrates the individual difference of RSV bioactivity and provides further support for nutritional intervention of RSV.

## Figures and Tables

**Table 1:** Primers of quantitative real-time polymerase chain reaction (qPCR).

Gene	Forward Primer (5′ to 3′)	Reverse Primer (5′ to 3′)
*cd36*	GCCAAGCTATTGCGACATGA	GGCATTGGCTGGAAGAACAA
*fabp1*	TGGTCAGCTGTGGAAAGGAA	TCCTGGCTCTGCAATTGGTA
*slc27a5*	CGCGTAGGAGACCTGTACTT	AACACACTCCACCTCTCCAG
*cpt1a*	CATATTCAGGCAGCGAGAGC	AGGTTTGAGTTCCTCACGGT
*acadm*	GAAAGCTGCTAGTGGAGCAC	CTGGTAACTGAGCCTAGCGA
*pdk4*	GATGCTCTGTGCCTTTCCTG	TGCGACTCAGGCCTCATAAT
*pparg*	GGAAGACCACTCGCATTCCT	GTAATCAGCAACCATTGGGTCA
*abca1*	CAGGAAGCACGTGTCTGAAG	GTGGTCTCCGAGATGCCATA
*abcg5*	CCGCGGACTTCTACAACAAG	TGTGGTTGGCTCATCTAGCA
*abcg8*	GAAGGCACAGTCTCTTGCAG	AGTCAGTGTCCTGTGTGAGG
*ldlr*	GAACTCAGGGCCTCTGTCTG	AGCAGGCTGGATGTCTCTGT
*scarb1*	TTGGCCTGTTTGTTGGGATG	ATCGATCTTGCTGAGTCCGT
*gapdh*	AACGGATTTGGCCGTATTGG	CATTCTCGGCCTTGACTGTG

## Supplementary Materials

The following supporting information can be downloaded at https://www.mdpi.com/article/10.3390/foods12010094/s1. Table S1. The amplification efficiency of primers. Table S2. The chemical composition of diets. .Figure S1: Cytotoxicity assay of RSV and phenolic acids at doses from 0 to 200 μM with and without OA for 24 h.
