# Supplementary material for: Alleviation Effects of Microbial Metabolites from Resveratrol on Non-Alcoholic Fatty Liver Disease
Source: Foods. 2022 Dec 24;12(1):94. doi: 10.3390/foods12010094 (PMC9818778; doi:10.3390/foods12010094)

Supplemental Figure legends

Supplementary Table S1. The amplification efficiency of primers.

Gene	amplification efficiency (%)
cd36	94.31
fabp1	92.18
slc27a5	103.67
cpt1a	108.26
acadm	107.75
pdk4	98.33
pparg	95.91
abca1	105.36
abcg5	96.49
abcg8	93.15
ldlr	93.78
scarb1	103.42
gapdh	105.59

Supplementary Table S2. The chemical composition of diets

Ingredient	Normal diet (ND)		High-fat diet (HFD)	
	g%	kcal%	g%	kcal%
Casein, 30 Mesh	18.96	19.72	25.84	19.72
L-Cystine	0.28	0.30	0.39	0.30
Corn Starch	47.98	49.91	0.00	0.00
Maltodextrin 10	11.85	12.32	16.15	12.32
Sucrose	6.52	6.78	8.89	6.78
Cellulose, BW200	4.74	0.00	6.46	0.00
Soybean Oil	2.37	5.55	3.23	5.55
Lard*	1.90	4.44	31.66	54.35
Mineral Mix S10026	0.95	0.00	1.29	0.00
DiCalcium Phosphate	1.23	0.00	1.68	0.00
Calcium Carbonate	0.52	0.00	0.71	0.00
Potassium Citrate, 1 H2O	1.56	0.00	2.13	0.00
Vitamin Mix V10001	0.95	0.99	1.29	0.99
Choline Bitartrate	0.19	0.00	0.26	0.00
FD&C Yellow Dye	0.00	0.00	0.01	0.00
Energy (kcal/g diet)	3.85		5.24	

Supplementary Figure S1. Cytotoxicity assay of RSV and phenolic acids at doses from 0 to 200 μM with and without OA for 24 h. (A) RSV; (B) 3-HBA; (C) 3-HPP; (D) 4-HBA; (E) 4-HPA; (F) 4-HPP.

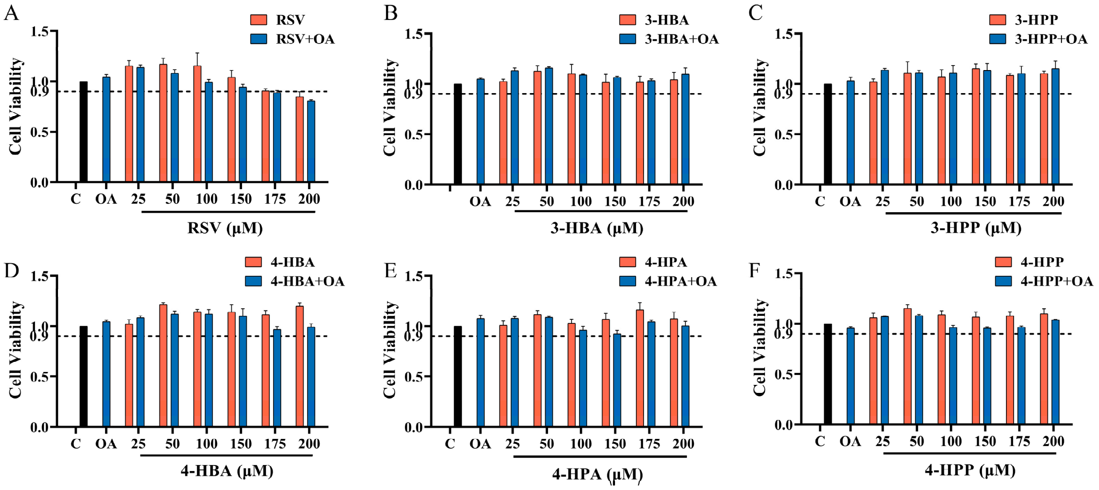

Supplement: Supplementary file 1 [file foods-12-00094-s001.zip › foods-2018236-supplementary.pdf]
